# Prevalence, causes, and staff perception of same-day elective surgery cancellation at a university hospital: a mixed study

**DOI:** 10.1186/s42506-025-00197-9

**Published:** 2025-09-15

**Authors:** Eman M. Nasr, Mahi M. Al Tehewy, Tarek Y. Youssef, Dalia A. Ibrahim, Shaymaa M. El-Bokl

**Affiliations:** 1 Department of Healthcare Quality, Faculty of Medicine, Ain Shams University, Cairo, Egypt; 2 Department of General Surgery, Faculty of Medicine, Ain Shams University, Cairo, Egypt; 3 Department of Anesthesia and Intensive Care, Faculty of Medicine, Ain Shams University, Cairo, Egypt

**Keywords:** Elective surgery, Cancellation, OR utilization

## Abstract

**Background:**

Same-day elective surgery cancellations disrupt healthcare efficiency by wasting resources and increasing costs due to repeated preparations and extended hospital stays. Beyond the financial toll, delays in necessary procedures can worsen health conditions, heighten patient anxiety, and prolong recovery times. These cancellations also place emotional and logistical burdens on patients and families, further amplifying their impact. This study aimed to determine the prevalence of same-day elective surgery cancellations at the El-Demerdash University Hospital in Egypt, identify the underlying causes—including hospital-related, patient-related, and staff-related factors—and explore the perceptions of operating room (OR) staff regarding these causes.

**Methods:**

A mixed-methods approach was used, combining quantitative and qualitative components. The quantitative component was a cross-sectional study including the prospective collection and analysis of 993 elective surgeries performed in 21 ORs at El-Demerdash University Hospital. The qualitative component involved 25 OR staff members and comprised two focus-group discussions with OR nurses, as well as 15 in-depth interviews with surgeons, anesthesiologists, and OR secretaries.

**Results:**

The cancellation rate for same-day elective surgeries was 12.59%. Key reasons for cancellation included unavailable OR time (24.03%), lack of ICU beds (21.71%), changes in the patient’s medical condition (14.73%), prolonged previous surgeries (10.85%), and equipment issues (7.75%). The qualitative analysis revealed that most OR staff expected a cancellation rate of 10–20%, identifying ICU bed unavailability and changes in patient condition as the primary contributing factors.

**Conclusion:**

Same-day elective surgery cancellations at El-Demerdash University Hospital are lower than the rates reported in most developing countries but remain higher than the international efficiency benchmark. The leading causes are primarily avoidable and hospital related. To improve OR utilization, strategic management interventions targeting the key causes are recommended. These include optimizing the OR booking system, improving department communication, and ensuring proper preoperative patient preparation and education and better resource allocation.

## Introduction

Elective surgery cancellation occurs when a scheduled surgical procedure is booked but not performed on the intended day [1]. Same-day surgery cancellations negatively impact operating room (OR) efficiency by reducing OR utilization, wasting resources, and contributing to significant financial losses for patients, hospitals, and the healthcare system [2]. A study from a large teaching hospital in Finland estimated the average cost of a single operation cancellation to be 2459.91 euros [3]. Beyond economic consequences, cancellations also have a significant psychological and social impact on patients and their families [4, 5].

In the literature, reported cancellation rates vary widely. A study from Brazil noted that cancellation rates in developed countries range from 0.37% to 28% [6], while other studies have reported even higher rates in developed nations, ranging from 2 to 40% [7–10]. In low- and middle-income countries, cancellation rates can reach as high as 73% [4, 11–13]. In Egypt, a study conducted in the Pediatric Surgery Department at a university hospital in Alexandria reported an average cancellation rate of 25% [14]. In 2021, a cross-sectional study by El Bokl and colleagues at the Cardiothoracic Ain-Shams University Hospital reported a cancellation rate of 21.7% [15]. These wide variations are often influenced by factors such as hospital settings, cultural context, and a country’s socioeconomic conditions [16, 17].

Previous studies have reported causes of surgery cancellations either by grouping them into broad categories or listing them individually without clear categorization [18]. Generally, these causes fall into three categories: patient-related factors (e.g., refusal to consent, acute infection), staff-related factors (e.g., staff unavailability), and hospital-related factors (e.g., lack of ICU beds, equipment issues, prolonged prior surgeries) [19]. Additionally, some causes are considered avoidable—such as equipment shortages—while others, such as sudden changes in a patient’s medical condition, are deemed unavoidable. Hospitals committed to quality improvement efforts aim to address avoidable causes in order to reduce costs, improve OR utilization, and enhance patient satisfaction [20].

In the study by El Bokl et al. [15], the most frequent cause of cancellations at the Cardiothoracic Ain-Shams University Hospital was the inclusion of standby operations, which accounted for 29.4% of all cancellations. Standby operations are cases scheduled (overbooked) and prepared to replace any other case that might be cancelled [15]. Cardiothoracic operations are complex, with long surgical and turnover time, and nearly all cases require postoperative ICU beds, which are factors that may contribute to higher cancellation rates. Similarly, Chiu et al., in a teaching hospital in Hong Kong, identified facility factors such as unavailable OR time [10]. Chang et al., in a 6-year retrospective study in a Chinese university hospital, pointed to scheduling errors and surgeon delays [5]. In the Middle East, Morris et al. linked cancellations to patient no-shows during a 1-month study in a newly opened hospital [7]. Additionally, a prospective observational study conducted over seven consecutive days in March 2017 across 245 British National Health Service hospitals reported that 33.3% of cancellations were due to changes in patients’ medical conditions, while 31% were attributed to insufficient bed capacity [21].

Elective surgery cancellations pose a significant challenge for healthcare systems worldwide, particularly in developing countries like Egypt. Given the country’s dense population and ongoing economic constraints, the efficient use of hospital resources is critical. Although the issue of cancellation has been previously examined in Egyptian studies [14, 15], those studies focused on highly specialized tertiary hospitals that did not include multiple surgical specialties. Furthermore, research addressing this problem in Egypt remains limited, and little is known about staff perception of the causes based on real-world data. The El-Demerdash University Hospital, as a major tertiary surgical center, provides an ideal setting to investigate this issue, where cancellation of elective surgeries may have a more profound effect. Thus, this study aims to measure the prevalence and identify the causes of elective surgery cancellations in a large, multispecialty tertiary surgery hospital. Moreover, it aims to explore staff perception of these causes. By providing this information to healthcare managers and policymakers, the study hopes to support the development and implementation of effective strategies to minimize cancellations and improve resource utilization.

## Methods

### Study question

- What is the prevalence of same-day elective surgery cancellation in a large, multispecialty tertiary surgery hospital?

### Study design

A mixed approach design (sequential explanatory design) was adopted, comprising the following:

- *Quantitative component*: A cross-sectional study was conducted to measure the prevalence of and identify the causes behind the cancellation of elective surgical procedures.
- *Qualitative component*: Focus-group discussions and in-depth interviews were used to explore the perceptions of OR staff regarding the reasons for elective surgery cancellations. This approach was preferred over surveys due to its flexibility, allowing deeper exploration based on participants’ answers.

This sequential explanatory design enhances the study’s validity by contextualizing statistical trends with real-world insights. The quantitative component identifies the prevalence and patterns of cancellations, while the qualitative component interprets these findings by exploring underlying reasons, staff perceptions, and system-level challenges from healthcare providers’ perspectives. This mixed-methods approach is particularly suited to investigating complex, multifactorial issues such as surgical cancellations and strengthens validity through methodological triangulation [22].

### Study setting

This study was conducted at El-Demerdash University Hospital, a tertiary care facility with a capacity of 477 beds in Cairo, Egypt. The hospital’s operating department consists of 21 operating rooms (ORs) dedicated to elective surgeries, covering both general and specialized procedures. General surgery departments include upper gastrointestinal, lower gastrointestinal, hepatobiliary, breast, endocrine, and bariatric surgeries. Specialized surgery departments include orthopedic, ENT, plastic, urology, neurosurgery, and vascular surgeries. The ORs operate 5 days a week (Saturday to Wednesday) from 8:00 AM to 8:00 PM. Thursdays are reserved for private cases. A separate emergency theater, which functions 24 h a day, 7 days a week, was excluded from this study.

### Study duration

Quantitative data were collected from June 2022 to October 2022, while the qualitative study was conducted between September and December 2023.

### Sample size and technique

**Fig. 1:**
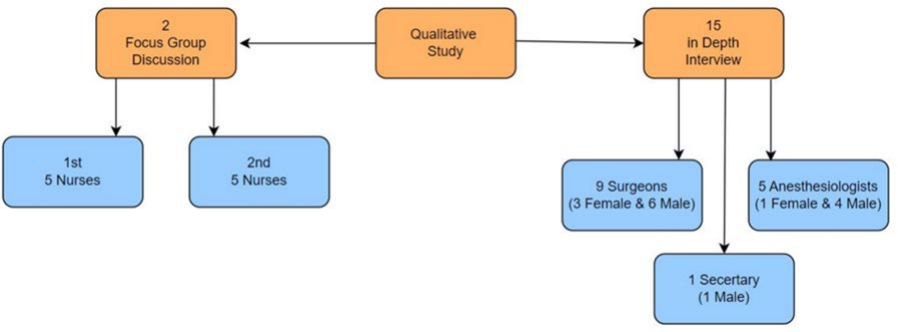
Attendees of the qualitative study

- *For the quantitative study*, PASS 11 software was used for sample size calculation as it is a powerful, reliable, and versatile tool supporting a wide range of statistical tests. The sample size was calculated at a 95% confidence level, with a margin of error of ± 5% for estimating the cancellation rate and ± 10% for identifying cancellation causes. Based on previous studies, a minimum of 350 elective operations was required to estimate the cancellation rate, and at least 74 canceled surgeries were required to identify causes. Ultimately, data collection included 125 canceled surgeries out of a total of 933 elective operations. A convenience sampling method was used to allow for prospective data collection and to enable cross-checking of cancellation causes with the surgeons. To mitigate potential selection bias inherent in convenience sampling, the operating department was visited on alternating days, and all the surgical lists and operations on these days were included in the study. Additionally, all working days of the week were represented in the sample.
- *The qualitative study* included 25 OR staff members (Fig. 1), selected through purposive sampling. Data collection continued until saturation was reached—that is, when no new themes or patterns emerged from the interviews and focus-group discussions [23, 24].

### Data collection

#### Quantitative data

Standardized data collection sheets were designed to gather information on all scheduled and canceled surgeries. Reasons for cancellation were initially extracted from the surgical logbook, then verified by contacting the respective surgeons. Surgeons were asked to state the reason for cancellation without being prompted with the recorded entry, to ensure accuracy. When necessary demographic data (e.g., age, residency) or medical information (e.g., diagnosis, procedure) was missing from the surgical lists, the hospital’s IT system was accessed using patient names and unique identification numbers. Medical records were also reviewed to calculate American Society of Anesthesiologists (ASA) physical status scores [25] for canceled cases, based on the patient’s preoperative anesthetic assessment. Cancellation causes were categorized into hospital-related, staff-related, or patient-related factors. Initial categorization was performed by one researcher following the classifications used in similar studies. Any ambiguous cases were further reviewed and categorized by consensus among all authors. A canceled case was defined as an elective surgery that had been scheduled and submitted to the OR secretary by 7:00 AM but was subsequently canceled on the same day.

#### Qualitative data

A standardized moderator guide, developed by the researchers and reviewed by experts, was used for all interviews and focus-group discussions. The guide consisted of open-ended questions targeting and discussing key causes of surgical cancellations and required no modifications following pilot testing. To ensure reliability and minimize bias, the same researcher conducted all sessions using neutral probing and consistent facilitation. All sessions were conducted face to face, audio-recorded, and supplemented by real-time note-taking to enhance accuracy and transparency. Both methodological and data source triangulation were employed to strengthen credibility. Semi-structured interviews with surgeons and anesthetists captured individual perspectives, allowing for in-depth exploration of their specific roles and experiences. Focus-group discussions with nurses revealed shared team dynamics. This multi-perspective approach enriched the understanding of factors influencing OR cancellations and reinforced the trustworthiness of the analysis. Additionally, an expert in qualitative research guided the analysis process to support the dependability and confirmability of the findings, although independent coding was not performed.

### Statistical analysis

- *Quantitative analysis*: Data was cleaned and entered into Microsoft Excel before analysis using SPSS version 25 (IBM Corp., Armonk, NY, USA). Descriptive statistics were calculated, with means and standard deviations reported for quantitative variables and frequencies and percentages for qualitative variables. Group comparisons were performed using chi-square tests for categorical variables, and pairwise comparisons were adjusted using the Bonferroni correction. Also, logistic regression analysis was performed to identify predictors of cancellations.
- *Qualitative analysis*: Transcriptions from interviews and discussions were manually prepared and analyzed using a thematic analysis approach. The data were coded, and themes were identified using a “fracturing and grouping” method: data were initially broken into discrete segments (“fracturing”) and then reorganized into categories and overarching themes (“grouping”), based on recurring patterns and meanings. This followed a structured process of qualitative data interpretation [23].

## Results

### Quantitative study results

#### Demographic data

Out of 993 scheduled cases during the study period, 125 (12.59%) were cancelled on the same day of surgery. As shown in Table 1, the mean age of the participants was 40.5 ± 19.49 years, with the majority (66.26%) falling within the 25–64 age group. The distribution of gender and residence (within Cairo vs. outside Cairo) was nearly equal.

**Table 1:** Demographic data (age, gender, residence) of the studied cases in El-Demerdash Hospital during June to October 2022, Cairo, Egypt

Demographic data	*N* (%)
**Age (in years)#** Child (≤ 14) Youth (15–24) Adult (25–64) Senior (≥ 65)	Mean ± (SD) = 40.5 ± (19.49) Median = 41, *IQR* = 30
	107 (10.84) 110 (11.15) 654 (66.26) 116 (11.75)
**Gender** Male Female	519 (52.27) 474 (47.73)
**Residence@** Cairo Outside Cairo	498 (50.25) 493 (49.75)
**Total**	993 (100)

^#^Age categories, life cycle groupings according to Canada statistics [26]. Number of missing data in this variable = 6

@Number of missing data in this categorical variable = 2

#### Cancellation in relation to independent data

In Table 2., the highest cancellation rates were observed among patients aged 65 and above (22.4%), followed by children aged 14 and under (14%). Age was the only demographic factor that showed a statistically significant difference between canceled and non-cancelled cases. A pairwise comparison using the Bonferroni correction indicated that the difference was driven by the senior age group. In contrast, gender and residence did not show statistically significant differences.

**Table 2:** Demographic characteristics among cancelled and non-cancelled cases in El-Demerdash Hospital during June to October 2022, Cairo, Egypt

Demographic data	Cancelled	Non-cancelled	Test value *χ*^2^	*p*-value*
	*N* (%)	*N* (%)		
**Age# (in years)** Child (≤ (1 Youth (15–24) Adult (25–64) Senior) ≥ (65	15 (14.02) 10 (9.09) 72 (11.01) 26 (22.41)	92 (85.98) 100 (90.91) 582 (88.99) 90 (77.59)	13.180	**0.004**
**Gender** Male Female	67 (12.91) 58 (12.24)	452 (87.09) 416 (87.76)	0.102	0.749
**Residence@** Cairo Outside Cairo	61 (12.25) 62 (12.58)	437 (87.75) 431 (87.42)	0.024	0.876
Total	123	868		

#Age categories, life cycle groupings according to Canada statistics [26]. Number of missing data in this variable = 6

@Number of missing data in this categorical variable = 2 cancelled cases

*Statistical significance at p-value ≤ 0.05

Table 3 reveals that vascular surgery had the highest cancellation rate (25.40%), followed by plastic surgery (16.0%) and urological surgery (14.53%). There was a statistically significant difference in cancellation rates across surgical departments. Post hoc pairwise comparisons using the Bonferroni correction revealed that differences between departments—such as general surgery vs. vascular surgery, urology vs. ENT, and plastic surgery vs. neurosurgery—contributed to this significance. In addition, the pain management department recorded zero cancellations. It is worth noting that 20% of cancellations occurred after the patient had already arrived at the operating department.

**Table 3:** Distribution of cancelled and non-cancelled cases according to surgical departments in El-Demerdash Hospital during June to October 2022, Cairo, Egypt

Surgical data		Cancelled	Non-cancelled	Test value *χ*^2^	*p*-value*
		*N* (%)	*N* (%)		
**Department**	Vascular surgery	16 (25.40%)	47 (74.60%)	16.644	**0.020**
	Plastic surgery	16 (16.00%)	84 (84.00%)		
	Uro-surgery	18 (14.53%)	144 (85.47%)		
	Neurosurgery	11 (12.94%)	74 (87.06%)		
	Orthopedic surgery	16 (11.27%)	126 (88.73%)		
	ENT	17 (11.11%)	100 (88.89%)		
	General surgery#	31 (10.47%)	265 (89.53%)		
	Pain management	0 (0.00%)	28 (100.00%)		
Total		125	868		

^#^General surgery includes upper GIT, lower GIT, hepatobiliary system, breast surgery, endocrine surgery, and bariatric surgery

^*^Statistical significance at *p*-value ≤ 0.05

#### Multivariate analysis

As shown in Table 4, logistic regression analysis identified age and surgical department as significant factors influencing OR cancellations. Patients aged 65 or older, as well as those scheduled for vascular surgeries, had more than twice the risk of cancellation compared to other age groups and surgical specialties. Some odds ratios showed relatively wide confidence intervals, suggesting data variability. Although the value of Nagelkerke *R*^2^ was low (0.053), Hosmer and Lemeshow test indicated a good model fit, with a *p*-value of 0.593.

**Table 4:** Logistic regression analysis of the studied demographic and surgical data in El-Demerdash Hospital during June to October 2022, Cairo, Egypt

**Variable**		***B* (coefficient of regression)**	*p*-value*	**Odds ratio Exp(B)**	**95% confidence interval for Exp(B)**
	Youth age group is the reference category				
**Age**	Child	0.455	0.356	1.575	(0.600)–(4.140)
	Adult	0.227	0.578	1.255	(0.565)–(2.788)
	Senior	0.976	.037*	2.654	(1.059)–(6.650)
**Gender**	Male is the reference category				
	Female	.027	0.906	1.028	(0.652)–(1.620)
**Residence**	Cairo is the reference category				
	Outside Cairo	0.199	0.373	1.221	(0.787)–(1.893)
**Departments**	General surgery is the reference category				
	Orthopedic surgery	− 0.237	0.560	0.789	(0.355)–(1.753)
	Uro-surgery	0.064	0.865	1.066	(0.512)–(2.218)
	ENT	0.145	0.734	1.156	(0.502)–(2.659)
	Plastic surgery	0.333	0.432	1.395	(0.608)–(3.199)
	Vascular surgery	1.004	.015*	2.728	(1.213)–(6.139)
	Neurosurgery	− 0.104	0.829	0.901	(0.351)–(2.318)
	Pain management	− 18.554	0.998	0.000	0.000
**Type of surgery**	Minor and moderate surgeries are reference categories				
	Major surgery	0.153	0.766	1.166	(0.426)–(3.191)
	Surgery requiring skills	0.602	0.196	1.826	(0.732)–(4.554)
	Advanced surgery	0.264	0.571	1.302	(0.523)–(3.243)

^*^Statistical significance at *p*-value ≤ 0.05

#### Causes of cancellation

**Fig. 2:**
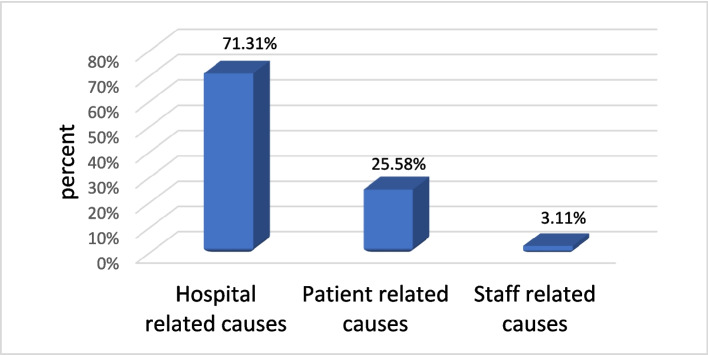
Causes of cancellation according to the cancellation category in El-Demerdash Hospital during June to October 2022

**Fig. 3:**
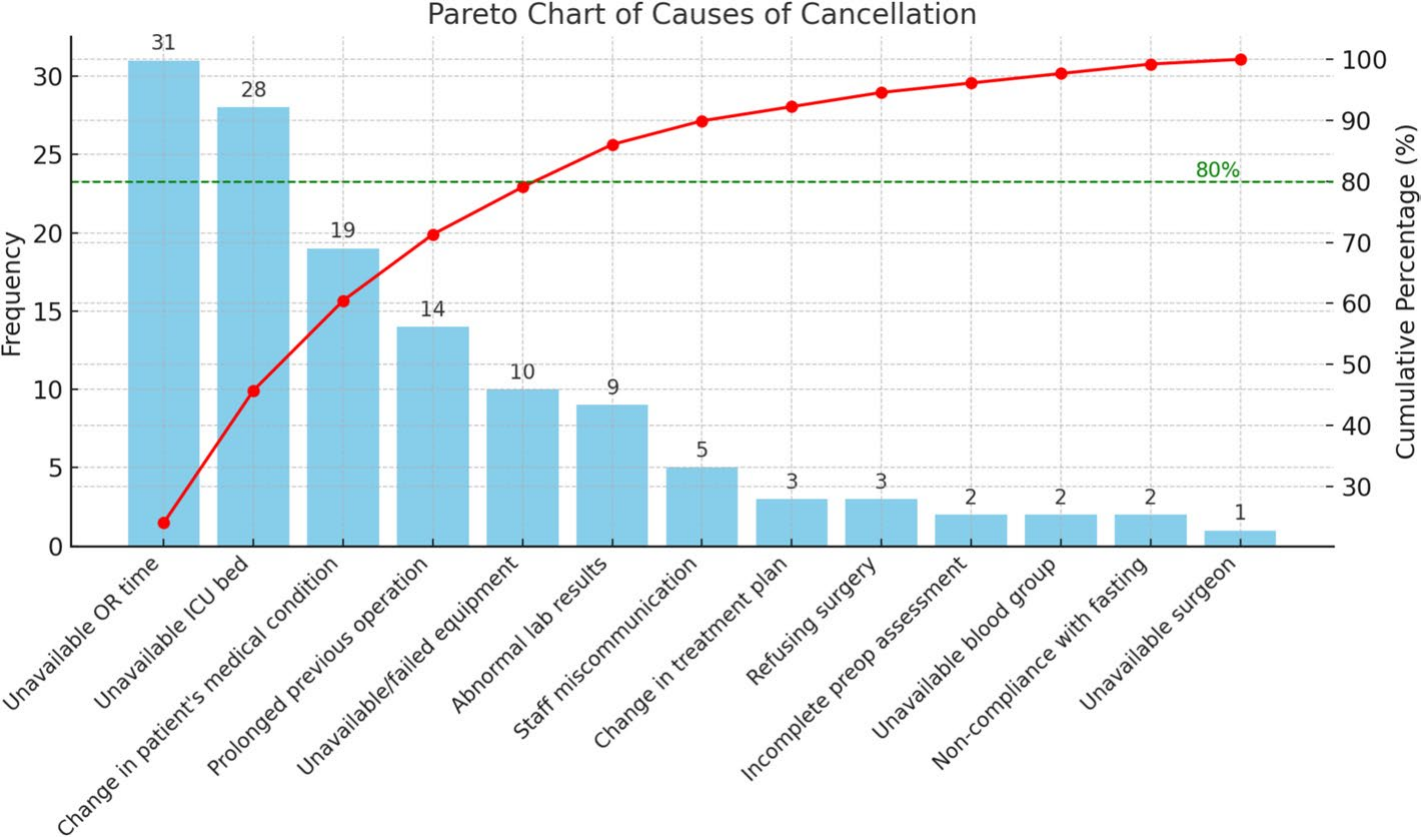
Pareto chart showing the vital few causes of cancellation in El-Demerdash Hospital during June to October 2022

The majority of same-day elective surgery cancellations were attributed to hospital-related factors, accounting for more than two-thirds of all cancellations (Fig. 2). A Pareto chart (Fig. 3) highlighted five key causes responsible for 80% of these cancellations, with unavailable OR time being the most common cause (24.03%), followed by unavailable ICU beds (21.71%), changes in the patient’s medical condition (14.73%), prolonged previous operations (10.85%), and unavailable/failed equipment (7.75%). The least frequent cause was the unavailability of the surgeon, accounting for only 0.78% of cancellations.

**Fig. 4:**
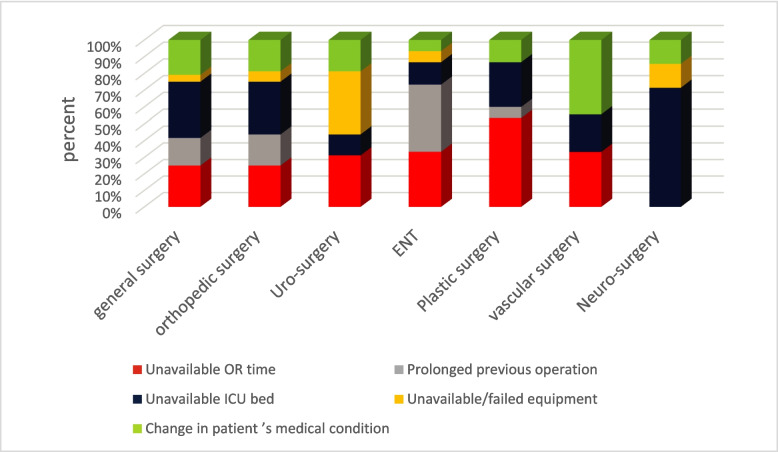
Stacked bar chart representing the distribution of the vital few causes of cancellation among surgical specialties in El-Demerdash Hospital during June to October 2022

Figure 4 illustrates the distribution of the major causes of cancellation across different surgical specialties. Unavailable OR time, the most prevalent cause, affected all departments except neurosurgery and accounted for nearly half of the cancellations in the plastic surgery department. Unavailable ICU beds, the second most frequent cause, impacted all departments and were the leading cause of cancellations in neurosurgery. Prolonged previous operations were most commonly reported in the ENT department, while failed equipment was the leading cause of cancellation in urological surgery.

**Fig. 5:**
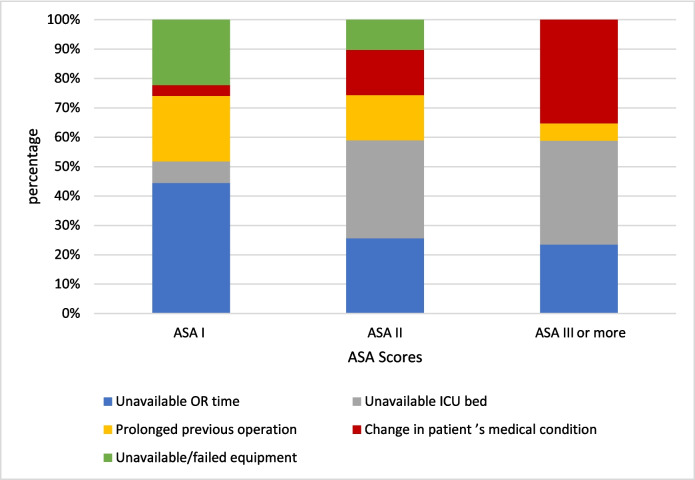
Stacked bar chart representing the distribution of the vital few causes of cancellation based on the American Society of Anesthesiologists (ASA) physical status classification [25]

The physical status scores of cancelled cases were determined according to the American Society of Anesthesiologists (ASA) classification system [25]. Among the cancelled cases, 40.98% were classified as ASA II, 35.25% as ASA III or higher, and 23.77% as ASA I. Notably, more than 92% of cancellations caused by unavailable ICU beds involved patients classified as ASA II or III and above. Additionally, 85.8% of cancellations due to prolonged previous operations involved patients with ASA I or II scores. Conversely, 63.2% of cancellations related to changes in the patient’s medical condition involved ASA II or III and above patients. These differences were statistically significant (*p* < 0.05), as shown in Fig. 5.

### Qualitative study results

Two focus-group discussions and 15 in-depth interviews were conducted as illustrated in Fig. 1.

- *Staff perception of cancellation rate*: Most OR staff estimated that the elective surgery cancellation rate at El-Demerdash Hospital ranges from 10 to 20%. A few, primarily anesthesiologists, believed the rate to be less than 5%. Interestingly, most anesthesiologists remarked that the actual cancellation rate was higher than anticipated.
- *Staff perception of hypothetical common causes of elective surgery cancellation*: Half of the OR staff identified unavailable ICU beds as the primary cause of cancellations, followed by changes in the patient’s medical condition. A few, mostly anesthesiologists, attributed cancellations to patient-related factors, such as illiteracy. One anesthesiologist commented, “A patient may forget to report an important issue related to their medical condition, which is only discovered right before surgery or while on the operating table, leading to cancellation.”
- *Staff perception of the real causes of elective surgery cancellation:*
  - ◦ ***Unavailable OR time and prolonged previous operation***: Most surgeons and anesthesiologists agreed that these were major causes, while many nurses expressed surprise. Over half of the participants attributed these causes to intraoperative delays stemming from intraoperative unexpected complications or the longer time required by junior/trainee staff in teaching hospitals. Some, particularly surgeons, blamed inefficient time management, such as prolonged turnover time, and nearly half cited scheduling errors, including overscheduling and logistical constraints like anesthesia deadlines.
  - ◦ ***Unavailable ICU beds***: Most staff agreed this was a critical issue, attributing the shortage to emergencies or postoperative cases occupying ICU beds, creating scheduling uncertainties. Some surgeons highlighted scheduling multiple complex cases requiring ICU admissions on the same list as a contributing factor. One surgeon also pointed to poor communication between anesthesiologists and ICU staff as another contributing factor.
  - ◦ ***Patient’s medical condition changes***: Many participants agreed that cancellations sometimes result from preoperative stress triggering unexpected changes in vital signs, even in previously stable patients. Others highlighted issues such as incomplete preoperative preparation or missed medication doses as potential contributors. A few staff members disagreed with this cause being significant.
  - ◦ ***Unavailability/equipment failure***: Approximately half of the staff, primarily surgeons and nurses, identified this as a cause. They attributed it to delays or incorrect equipment deliveries by suppliers. They also pointed to the challenges in detecting such errors due to sterilization concerns. Additionally, staff highlighted that scheduling multiple cases requiring the same equipment at the same time often led to cancellations.

## Discussion

Cancellation of elective surgeries is a key indicator of healthcare quality, with significant implications for both patient care and hospital management. Not only it does reflect inefficiencies in hospital operations but also it carries substantial cost implications and adversely affects patient satisfaction.

### Overall cancellation rate and comparison to literature

This study revealed that the overall same-day elective surgery cancellation rate at El-Demerdash University Hospital was 12.59%. This rate is notably lower than that reported in a comparable Egyptian study by El Bokl et al. (2021) at the Cardiothoracic Ain-Shams University Hospital, which recorded a cancellation rate of 21.7% [15]. The latter hospital is a highly specialized facility that manages complex patients and procedures, which likely contributes to its higher cancellation rate, in addition to its practice of preparing standby patients in anticipation of possible cancellations [15]. The cancellation rate observed in the current study was also lower than that reported in several other low- and middle-income countries. For instance, in India, a large multidisciplinary government hospital reported a same-day cancellation rate of 17.6% [27]. One possible explanation for this discrepancy is the difference in scheduling systems: in the Indian study, operative lists were finalized at 14:00 the day before surgery, whereas in our study, final lists were submitted at 7:00 a.m. on the same day, potentially allowing for more up-to-date decision-making and fewer cancellations. In other developing countries, the rates were even higher: 18.45% in Ethiopia [28], 21.41% in Brazil [29], and 48.5% in Nigeria [30]. The high rates in Ethiopia and Nigeria may reflect limitations in healthcare infrastructure and resource availability. Overall, surgical cancellations are complex and multifactorial, with discrepancies influenced by differences in patient populations, case complexity, hospital settings, and operating room management practices [13, 28].

Conversely, the cancellation rate reported in this study exceeds the commonly cited efficiency benchmark of approximately 5% [7, 31]. It also exceeds the rates observed in Jordan (3.6%) [33] and high-income countries, including Hong Kong (7.6%) [10], Saudi Arabia (11.1%) [32], and the UK (1.1%) [34]. According to Schuster et al. (2011), university hospitals tend to have higher cancellation rates than smaller hospitals due to less predictable workflows, the demands of teaching environment, higher patient loads, and greater complexity of cases [35]. As a major tertiary referral center serving multiple surgical specialties, El-Demerdash University Hospital shares these characteristics, which may explain its relatively high cancellation rate. Interestingly, the observed cancellation rate closely matched the perceptions of most OR staff interviewed in the qualitative component of this study. Most staff estimated a cancellation rate between 10 and 20%, while anesthesiologists tended to estimate lower rates (less than 5%), likely due to varying interpretations of what constitutes a cancelled surgery. Specifically, many staff members considered a surgery “cancelled” only if the patient had already arrived at the operating department, reflecting a possible misconception about the broader definition of surgical cancellation.

### Key demographic and departmental insights

Identifying the demographic groups most affected by cancellations can help guide targeted improvement efforts. In the current study, the mean age of patients was 40.5 years (± 19.49), with age showing a statistically significant difference between cancelled and non-cancelled cases. The highest cancellation rates were observed among seniors (aged 65 and older), likely due to their higher prevalence of comorbidities and increased need for postoperative ICU beds. This finding aligns with a study from a Korean university hospital, where 21% of surgeries in patients aged 60 or older were cancelled [36].

The current study also revealed significant differences in cancellation rates between surgical departments. Vascular surgery had the highest cancellation rate (25.40%), followed by plastic surgery (16%) and urology (14.53%). The high rate in the vascular department may be attributed to the medical complexity of patients, the higher proportion of elder patients, and a shortage of dedicated operating rooms, as only 1 out of 21 ORs was allocated for vascular surgeries. This contrasts with findings from a study in the UK, where the highest cancellation rates were observed in the ENT and general surgery departments, largely due to secretarial scheduling practices [37]. Notably, pain management experienced zero cancellation, possibly because these are performed solely by anesthetists, streamlining the process. An alternative explanation could be the small sample size of pain management cases included in this study.

### Vital few causes of surgical cancellations

Hospital-related factors accounted for the majority of cancellations (71.31%), followed by patient-related factors (25.58%). This finding is consistent with two systematic reviews, both of which identified hospital-related reasons as the most common cause of surgical cancellations [28, 38].

Regarding the OR staff’s expectations of the most common causes of cancellation, about half reported unavailable ICU bed as the leading cause of elective surgery cancellations. The quantitative analysis, however, identified this as the second most frequent cause, with changes in the patient’s medical condition ranking third. A few staff members attributed cancellations to patient-related factors, such as illiteracy. Although the hypothetical causes align with the findings of the quantitative study, none of the staff mentioned the actual leading cause “unavailable OR time.” Furthermore, the order of perceived causes did not match the actual rankings found in the data.

The most frequent hospital-related cause was unavailable OR time, responsible for 24.03% of cancellations. This could be due to various reasons, including anesthetic restrictions (e.g., a 3 p.m. cutoff for major surgeries), overscheduling, and prolonged previous operations. Similar findings were reported in a study from India, where 78% of cancellations were attributed to OR time constraints due to anesthetic restrictions [39]. Another study in Spain reported that 22.5% of cancellations were caused by a lack of OR time [40].

The qualitative findings from this study provided deeper insights into this issue. Many staff members attributed prolonged surgeries to intraoperative complications, the involvement of trainee surgeons, or inefficient time management. While surgeons often viewed the 3 p.m. anesthesia cutoff as a limiting constraint, anesthesiologists generally considered it a necessary safeguard to avoid overrunning scheduled hours.

In teaching hospitals such as El-Demerdash, surgeries often take longer due to the involvement of trainees, which contributes to surgical list overruns—a common challenge faced by academic institutions worldwide [37, 41]. Key strategies to reduce cancellations stemming from this issue include optimizing OR scheduling by adopting clear scheduling criteria and implementing mathematical modeling-based algorithms. Ensuring that all cases undergo preoperative anesthesia assessment and aligning surgical lists with available resources— such as staff, ICU beds, and equipment—are also essential. Emphasis should be placed on effective time management, particularly ensuring a timely start of the first case. Additionally, it is important to re-evaluate the 3:00 p.m. anesthesia cut-off policy, considering its advantages and drawbacks. Finally, linking staff incentives to performance in reducing cancellations and improving workflow efficiency may contribute to sustained improvements. The second most common cause of cancellation was the unavailability of ICU beds, accounting for 21.71% of all cancellations. Qualitative findings suggested several contributing factors: ICU bed availability was often confirmed on the same day of surgery, multiple complex cases requiring postoperative ICU care were sometimes scheduled on the same list, and poor communication between surgical, anesthetic, and ICU teams. Although our data cover only a specific data collection period, this issue appears to be ongoing rather than seasonal, likely reflecting the hospital’s status as a major tertiary referral center that handles a high volume of complex surgical cases each month. A UK study found that 66% of surgical cancellations in a teaching hospital were due to bed shortages, exacerbated by emergency admissions and medical patients requiring critical care [42].

In contrast, a study from Spain reported that cancellations due to bed shortages were rare, thanks to efficient preoperative planning and flexible bed allocation [40]. In our setting, suggested solutions include the following: improving interdepartmental, establishing clear criteria for postoperative ICU bed requests, using conventional or artificial intelligence (AI)-based prediction models to anticipate ICU needs, and increasing the number of dedicated ICU for elective cases.

Changes in the patient’s medical condition accounted for 14.73% of cancellations and were the leading cause among patient-related factors. These cancellations may have resulted from discrepancies between the preoperative assessments conducted the day before surgery and the clinical evaluations made by the operating room team on the day of surgery. A similar issue was reported in Spain, where 50% of cancellations were due to medical deterioration between the preoperative check and the operation [40]. Strategies to tackle this issue include providing counseling and education to reduce pre-surgical anxiety, ensuring adherence to medication and vital sign monitoring, and aligning anesthesia staffing levels with the surgical schedule to allow for comprehensive patient assessments. Additionally, optimizing the time between assessment and surgery may improve the accuracy of preoperative fitness assessments, especially for high-risk patients (ASA ≥ 3). Regular interdisciplinary meetings are also recommended to develop evidence-based policies aimed at reducing cancellations.

Unavailable or failed equipment contributed to 7.75% of cancellations, with a striking 60% of these cases occurring in the urology department. This issue was attributed to limited hospital resources, outdated equipment, and errors by external vendors responsible for providing specialized surgical tools. Equipment-related cancellations were also observed in other studies—for instance, a 4.2% equipment-related cancellation rate was documented in Saudi Arabia [43], while the UK reported a much lower rate of 0.4% [44]. To reduce such cancellations, implementing a comprehensive checklist to verify the availability and functionality of required equipment as part of the preoperative preparation process is strongly recommended. Furthermore, establishing contracts with reliable suppliers committed to delivering high-quality, timely equipment, along with continuous supplier evaluation, can significantly minimize the risk of last-minute equipment failures or shortages. Ensuring accountability and consistency in the surgical supply chain is essential for improving operating room efficiency and patient safety.

### Successful stories

A quality improvement project at a large tertiary care children’s cancer center in Riyadh, Saudi Arabia, succeeded in reducing operating room cancellations by 17%, lowering the rate from a baseline of 22% through a series of targeted interventions. A daily OR huddle was implemented to identify critical areas requiring intervention. These included personalized medical planning, pre-anesthesia evaluations, specialized consultations, and coordination of blood product support. Improvements in communication were also central to the project’s success, incorporating patient and nurse education, re-evaluation protocols, and a preoperative checklist review to ensure a 24-h cancellation notice to the OR scheduling team [45].

### Strengths and limitations

This study has several limitations. First, the study employed a cross-sectional design, which is appropriate for measuring prevalence but does not establish causality. Second, the study was conducted in a single, large surgical tertiary hospital (El-Demerdash Hospital), which may limit the generalizability of findings to other healthcare settings with different contexts. To enhance generalizability, future research in diverse hospitals and regions is recommended. Third, the use of convenience sampling may introduce selection bias. Although random sampling would provide more valid, generalizable results, it was not feasible in the current study where operative lists are generated day by day. However, measures were taken to minimize selection bias, as detailed in the sampling section. Moreover, surgical classifications were based on the hospital’s financial categorization rather than purely clinical distinctions. Additionally, the ASA classification scores were calculated only for cancelled cases, preventing direct comparison between cancelled and non-cancelled cases regarding that factor. No data were available on whether patients attended the anesthesia clinic, which limited the ability to assess any correlation between preoperative anesthesia evaluation and cancellations due to changes in the patient’s medical condition. Despite these limitations, the study has several notable strengths. The mixed-methods design provided a comprehensive understanding of the problem by integrating quantitative data with in-depth qualitative insights. Moreover, the large sample size enhanced the statistical robustness, and the prospective nature of data collection helped ensure its accuracy.

## Conclusion

The prevalence of same-day elective surgery cancellation at El-Demerdash University Hospital was considerable and primarily driven by hospital-related factors—most of which are potentially preventable. Addressing these challenges requires strategic enhancements in OR utilization management: improving OR booking system as a priority solution, improving communication between surgical and ICU teams, and integrating AI-enhanced tools for OR and ICU booking. Additionally, ensuring comprehensive preoperative patient preparation and education, along with improved resource allocation, is essential. Implementing these targeted interventions could significantly reduce cancellation rates and improve overall patient care. Future research is warranted to explore patterns of ICU utilization and to investigate the predictive role of ASA score in OR cancellations.

## Data Availability

The datasets used and/or analyzed during the current study are available from the corresponding author upon reasonable request.

## Abbreviations

AI: Artificial intelligence
ANOVA: Analysis of variance
ASA: American Society of Anesthesiologists
ICU: Intensive care units
IT: Information technology
OR: Operating room
SPSS: Statistical Package for the Social Sciences
